# Hybrid Model Based on Genetic Algorithms and SVM Applied to Variable Selection within Fruit Juice Classification

**DOI:** 10.1155/2013/982438

**Published:** 2013-12-10

**Authors:** C. Fernandez-Lozano, C. Canto, M. Gestal, J. M. Andrade-Garda, J. R. Rabuñal, J. Dorado, A. Pazos

**Affiliations:** ^1^Information and Communications Technologies Department, Faculty of Computer Science, University of A Coruña, Campus Elviña s/n, 15071, A Coruña, Spain; ^2^Analytical Chemistry Department, Faculty of Sciences, University of A Coruña, Campus da Zapateira s/n, 15008, A Coruña, Spain

## Abstract

Given the background of the use of Neural Networks in problems of apple juice classification, this paper aim at implementing a newly developed method in the field of machine learning: the Support Vector Machines (SVM). Therefore, a hybrid model that combines genetic algorithms and support vector machines is suggested in such a way that, when using SVM as a fitness function of the Genetic Algorithm (GA), the most representative variables for a specific classification problem can be selected.

## 1. Introduction

The evolution of technology has made accessible a wide range of information in almost any area of human activity. The increased connectivity and the development of Internet have allowed access to large volumes of data. Moreover, the increase of the processing capacity of computers, as well as the current low cost of computer storage, allows preserving up to the last generated byte.

However, even having the means necessary for the data preservation, it is common to find a large number of irrelevant information when solving a problem. Only a fraction of the data has different and significant profiles, whereas the rest is redundant information, unwanted noise, or worse, information captured or stored incorrectly. In recent years, various variable selection methods have been developed with the aim of mitigating these problems as far as possible [1].

The feature selection or extraction is first introduced in the early 1960s, becoming a research topic widely studied and used over the last four decades [1–3]. The need for these techniques arises, as noted, from the emergence of certain predictive variables that may become irrelevant or redundant. A predictive variable is considered irrelevant if its content does not provide any information that could clear uncertainty about the original set. Similarly, it is redundant when its value can be determined from other predictive variables. The variable selection methods are aimed at detecting redundant and/or trivial variables for a given problem. The purpose of using these techniques is to identify an “optimal” subset, made up of a minimum number of variables necessary to find a valid solution to a problem.

As a result of applying these methods, some benefits are obtained, consolidating their position throughout time [4–6]. Among others, it is worth mentioning that the variable selection methods allow decreasing the number of samples required to obtain optimum results in virtually any classification or clustering problem. This leads, for example, to the creation of a faster prediction model when it comes to analyzing a lower number of variables. Similarly, cost reduction is obtained, taking into account a temporal perspective as well as the one regarding the system complexity for data acquisition, since we are dealing with a smaller amount of information. As a result, the complexity of the problem to be solved is reduced.

In this paper, we propose the use of a prediction hybrid system combining GA and SVM. First, we will briefly mention the most representative selection techniques in recent times. Then, we will describe the methods used to develop the proposed system. Next, we will explain concisely the developed model, as well as the results obtained with it. These results, related to the classification of juice samples according to their sugar concentration, will be compared to those obtained in previous studies from the use of ANN, allowing a comparative study of both methods.

## 2. State of the Art

In recent years, the methodology used by different organizations has been forced to evolve due to the fact that storage and processing capacities have substantially increased. This progress has led researchers to develop a large number of selection and learning techniques that allow improving their working methods.

The variable selection techniques have become a very useful tool. Among the existing techniques it is worth mentioning, as the most representative ones, Principal Component Analysis (PCA)—a mathematical method [7], and Partial Least Squares (PSA) [8], which behave similarly from the point of view of variable selection.

The main component analysis method is characterized as a statistical technique for the information synthesis or dimension reduction [7]. We are dealing with a linear algorithm whose operation is based on the correlation between variables. The aim of these methods is to minimize the number of variables as much as possible with the minimum loss of information. However, its use entails a series of drawbacks. The most important one comes from the set of variables obtained from their use, as these latter one do not belong to the initial set but are a linear combination of them.

On the other hand, PLS is a mathematical approach used to establish a model that relates the information from two different datasets. The idea is to look not only for the directions with a larger amount of information within the set of predictive variables but also to select those which have a stronger relationship with the variables to be predicted [8, 9]. This is why it is said that the PLS models are governed by a criterion of predictive ability rather than by the fitness of the model to the data.

The use of these or other mathematical methods involves a series of drawbacks that encourage the search for new techniques, aimed at reducing them. Among the common disadvantages of using these methods, it is worth mentioning two main drawbacks. On the one hand, it is necessary to have a broad knowledge of the field to be discussed, since, depending on certain features (linearity, interdependence, etc.) it will be possible to apply only some methods or others. On the other hand, such methods tend to have a single valid solution, not a set of solutions, so that they would not be efficient in problems in which there are several global solutions, or a global solution and local variables.

In several studies, evolutionary methods such as variable selection techniques were used [10, 11] due to the fact that they minimize the drawbacks of using the above-mentioned techniques. From the field of evolutionary computation and, more specifically, from the area of Gas, there has been a significant number of approaches towards variable selection methods.

That is why, in order to increase the reliability of the variable selection methods, we have opted for new strategies, among which we consider of great importance the hybrid methods that employ GAs and, on the one hand, prediction mechanisms, among which artificial neural networks (ANNs) are worth mentioning.

The ANNs are methods of nonparametric prediction-classification that allow obtaining a high degree of accuracy in many problems, especially those related to human knowledge [12–14]. They are based on a computational model established according to biological neural systems that try to simulate the operation of human brain neurons at small scale. The training of the weights of the ANN through traditional neuronal network learning methods has proven to be a very effective process. The creation of a hybrid model combining GAs and ANN allows the use of the former system as a guideline for improving the latter. As a result, GAs are employed to optimize the number of entries into the neural predictor classifier, thus decreasing the generalization error and consequently the size of the models.

There have been several studies that have opted for the combination of evolutionary methods with classification mechanisms [6, 13, 15–17]. However, even after having developed very efficient models, ANNs have the disadvantage of undergoing training processes on a strong stochastic basis, leading to nonrepeatability of the process. This is why many researchers prefer to choose new techniques based on robust statistical principles such as support vector machines (SVM) [18–23].

Developed by Vapnik [24, 25] and based on the statistical learning theory, the support vector machines are fast becoming one of the most used methods of prediction classification. Although their use is fairly recent, a considerable number of researchers have already reported states of the art of their performance in a variety of applications in pattern recognition, regression estimation, and prediction of time series. For example, the study carried out by Min et al. [26] combined the SVM and the GAs with the aim of predicting the business failure risk and avoiding bankruptcy, with a particular model being tested in predicting the crisis in Taiwan by Wu et al. [27]. Tan et al. [11], besides combining GAs and SVM, opted for the use of PLS for the identification of mitochondrial proteins. There are also some comparisons of SVM with regression methods [28]. Huerta et al. [29] combined a GA with SVM for gene selection and classification of microarray data. Venegas [30] used the SVM in the classification of academic texts according to their lexical-semantic content; in the study carried out by Donís et al. [31] the SVM were used to estimate the creep rupture stress of ferritic steel. Pérez et al. [32] applied the SVM to a typical classification problem of failure identification in distribution systems of electric power. In Fernandez-Lozano et al. [15, 16], a hybrid approach combines GAs and SVM for protein identification in two-dimensional gel electrophoresis images. Tong et al. [33] proposed a GA based ensemble SVM classifier built on gene pairs. A GA-based for solving two dual quadratic programming problems with a twin parametric-margin SVM in the primal space is proposed by Wang et al. [34]. Won et al. [35] combined a novel GA-SVM to find features from biological sequences. A particle swarm optimization (PSO) [36] to optimize a GA-SVM method to predict single nucleotide polymorphisms (SNPs) and to select tag SNPs as pointed by Ilhan and Tezel [37] or to solve the heating system planning problem is presented [38]. Zhang et al. combined PSO with SVM for classifying magnetic resonance imaging (MRI), brain images [39]. Ocak [40] combined a GA with a SVM in a medical decision support system for the evaluation of fetal wellbeing. Uzer et al. [41] combined an artificial bee colony algorithm with SVM for classification. A hybrid approach with SVM and microarray data is presented by Li et al. [42]. A cross-study comparison of classification methods including ANN and SVM for predicting metastasis in breast cancer is presented by Burton et al. [43]. Other approaches using evolutionary computation techniques are presented in [44, 45].

## 3. Methods

### 3.1. Selection of Variables

Currently, there are several variable selection techniques. One of the guidelines used for their classification is given according to the approach used: the indirect or filter approach on the one hand [46] and the direct or wrapper approach [47] on the other hand. The filter techniques select subsets of variables in a preprocessing step regardless of the classification problem. On the contrary, the wrapper methods use machine learning to assign a rating to the subsets of samples according to their predictive ability. There is a third group of techniques, called embedded, which perform the selection of variables during the very learning process or classification of samples.

Once the type of variable selection technique to be used is defined, it is necessary to establish a mechanism which allows carrying out the search for significant variables. Ideally, the selection should be performed in terms of the entire subset of variables that can be formed, but however, this would involve analyzing numerous combinations, with their corresponding computation time loss. That is why we use search strategies that provide results as close as possible to the overall optimum value. As shown in Section 3, different methods have been developed in order to explore the set of variables, among which the best known is the one based on the use of GAs [48, 49].

Several studies have proposed the combination of GAs and selection and prediction methods such as selection techniques and data prediction, respectively. One of the selection-prediction methods most used in these hybrid models is the ANNs [50]. However, these models are not free of drawbacks. One of the criticisms associated with the use of the ANNs is due to their “black-box” characteristic, as it is difficult to understand their internal operation and the process which leads them to determine the appropriate solution when it comes to a set of patterns. Another drawback of their use as a selection or prediction method, and perhaps the most important one, is the one concerning the exact nonreproducibility of their training (or at least of their difficulty) as a result of the stochastic process by which the weights are initialized. GAs are search techniques inspired by Darwinian Evolution and developed by Holland in the 1970s [51]. GAs for feature selection were first proposed by Siedlecki and Sklansky [52]. Many studies have been done on GA for feature selection since then [6, 53], concluding that GA is suitable for finding optimal solutions to large problems with more than 40 features to select from. GA for feature selection could be used in combination with a classifier such SVM, optimizing it.

Today, there are other prediction-selection techniques that allow making up for the shortcomings caused by the use of ANNs. This paper is aimed at emphasizing a method recently developed within the field of machine learning, the SVM. This is a classification and regression algorithm family which currently shows comparable or better results than those obtained with ANNs or other statistical models in problems of pattern recognition, prediction, classification, or data mining. From their inception to the present day, the SVM have evolved in such a way that they have become a successful tool when dealing with highly dimensional data.

### 3.2. Genetic Algorithms

The evolutionary computation (EC) has reintroduced concepts of evolution and genetics to solve problems, mainly those related to optimization tasks [48, 54]. However, it is also worth mentioning the important influence of the studies carried out by Turing and Samuel in the 1950s, “Can machines think?” and “How can computers learn to solve problems without being explicitly programmed?” [55, 56].

Broadly speaking, the EC methods are search and optimization techniques consisting of the application of heuristic rules based on principles of natural evolution. In other words, these are algorithms that look for solutions according to properties of genetics and evolution. Among these properties, it is worth mentioning the survival of the fittest individuals (which implies that once the best solutions to a problem are reached, they will keep being this way) and heterogeneity (we mean basic heterogeneity, so that algorithms could have numerous types of information when creating solutions).

#### 3.2.1. General Outline of Operation

**Figure 1:**
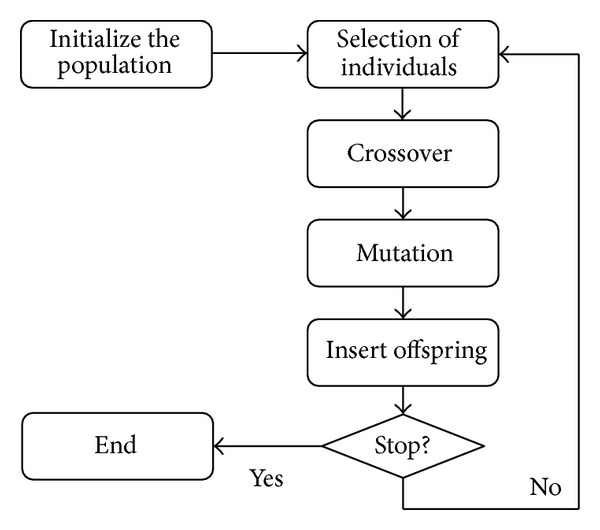
General outline of the operation of an evolutionary algorithm.

The evolutionary algorithms base their operation on a relatively simple outline, as shown in Figure 1. This iterative outline will refine solutions which will gradually be closer and closer to obtaining an overall solution of the problem.

But prior to the implementation of the evolutionary process specified by the algorithm, it is necessary to undertake two issues, perhaps the most important ones in the whole process: determining how to represent the solutions (encoding) and specifying a method which would allow us to know how good a solution is (fitness function).

One of the most used branches in the EC is made up of genetic algorithms (GAs) [49, 57]. In this case, the encoding of the solutions is performed through value chains (the chains being of fixed or variable length, with the values of these being bits, whole and real numbers, etc.).

The critical step when setting up an evolutionary algorithm is the definition of the fitness function. This function will have to evaluate every genetic individual, indicating a real value representing the goodness of the solution provided by the individual. This function will be responsible for guiding the search process in either direction. Precisely because we are dealing with a function responsible for verifying the goodness of each solution, this is an inherent aspect linked to the problem to be solved.

The above discussed evolution of solutions will happen due to crossover and mutation genetic operators which simulate processes of sexual and asexual reproduction that occur in a natural environment. Next, we will summarize each one of the remaining steps of which such algorithms consist.

*Initialization.* The power of evolutionary algorithms lies in the massively parallel exploration of the search space. This can occur due to the existence of numerous solutions, each exploring an area of the search space. The set of solutions, randomly initialized, is called genetic population.

*Selection.* The selection algorithms will be responsible for choosing the individuals which will and which will not have the opportunity to reproduce [58]. Since this is a simulation of what occurs in the natural environment, the fittest individuals have to be given more opportunities to reproduce. Therefore, the selection of an individual is related to its fitness value. However, the reproduction options of less fit individuals should not be completely eliminated, because in a few generations the population would become homogeneous in this way.

*Crossover. *Once the individuals are selected, they are recombined to produce offspring that will be inserted into the next generation. Their importance for the transition between generations is high because the usually employed crossover rates are around 90%.

The main idea of the crossover is based on that, if two individuals, properly adapted to their environment, are selected and the offspring obtained shares genetic information from both, there is a possibility that the inherited information is precisely the cause of their parents' goodness. By sharing the good features of two individuals, the offspring, or at least part of them, should have better characteristics than each parent separately.

*Mutation.* An individual's mutation causes that the value of one of its genes or nodes, usually only one of them varies randomly.

Although individuals can be selected directly from the current population and mutated before being introduced into the new population, the mutation is often used together with the crossover operator. Thus, the behavior that occurs in the natural environment is simulated, since when generating the offspring there is always some kind of error, usually with no consequence throughout the transmission of the genetic load from parents to offspring. The mutation causes sometimes a reduction in the individual's fitness value (which can be remedied in subsequent generations). However, the new information contributes directly to a significant increase of the goodness of the solutions or it can be a part of a better solution in future generations.

*Replacement.* The traditional operation of ANNs often includes the use of a temporary population. This latter is being filled by copying individuals and with the offspring generated due to crossover operations (and mutation, if that is the case). When this temporary population is complete—in this case it is said to have passed to a new generation—it becomes the population of the current generation, ruling out the previous one and repeating the process from a new empty temporary population. Such algorithms are usually called generational algorithms.

However, there is another approach, called steady-state algorithms. This option consists of working with a single population, which undergoes selections and insertions, ruling out the use of a temporary population. In this case, since the number of individuals in the population remains constant, it should be noted that a new individual cannot be added unless another is eliminated before that.

*Stopping Criterion.* As previously explained, the evolution process of solutions is essentially an iterative process. Therefore, it will be necessary to specify a criterion that allows establishing when the execution is completed. Once more, there are different options, but the most common ones are shown as follows.

- The fittest individuals in the population represent solutions good enough so that the problem could be solved.
- The population has converged. A gene has converged when 95% of the population has the same value (or a very similar one) for that gene. Once all the genes reach convergence it is said that the population has converged. When this phenomenon happens, the average goodness of the population is close to the goodness of the fittest individual.
- The difference of the best solutions found between different generations is reduced. This may indicate, at the very best, that the population has reached an overall solution or on the contrary that the population has come to a standstill at a local minimum value.
- A predetermined maximum number of generations have been reached.

It may be worth mentioning that the advantage of such techniques is the simplicity of their implementation. No technical knowledge is required to solve the problem, only one way that allows evaluating a possible solution (in order to define the fitness function). Moreover, it should be also noteworthy the simplicity of the ideas taken from the natural environment, on which the evolution of solutions is based.

In addition, this type of techniques is easily adaptable to multimodal problems (those with multiple solutions) [59] or multiobjective problems (those in which different criteria are optimized simultaneously) [60].

When the computational cost is a criterion to be considered due to its inherently parallel operation, we are dealing with easily distributable techniques (or at least the evaluation of the solutions, which often becomes a hurdle) with a marked improvement in the response time arising from such distribution.

Finally, we should note that these techniques, unlike others, always provide a solution to the problem raised and, in addition, this solution will be improving as implementation is carried out over time.

### 3.3. Support Vector Machines

The support vector machines are general methods for solving problems of classification, regression, and estimation. They are learning systems based on the studies performed by Vapnik on the statistical learning theory [24, 25]. From their inception to the present day, they have become the subject of continuous research and application. The interest raised by this method has increased considerably, becoming a referent for the other disciplines of machine learning and data mining.

At first, the SVM were developed to solve problems of binary classification (two classes), but currently, and throughout their evolution, they have widened their field of action, dealing with any kind of problems. The SVM are aimed at finding a linear optimal hyperplane distributing the data into two or more classes, so that all those elements which belong to the same class are located on the same side. This is equivalent to solving a classical quadratic programming problem, which guarantees the existence of a single solution and a reasonable efficiency for real problems with thousands of examples and attributes.

**Figure 2:**
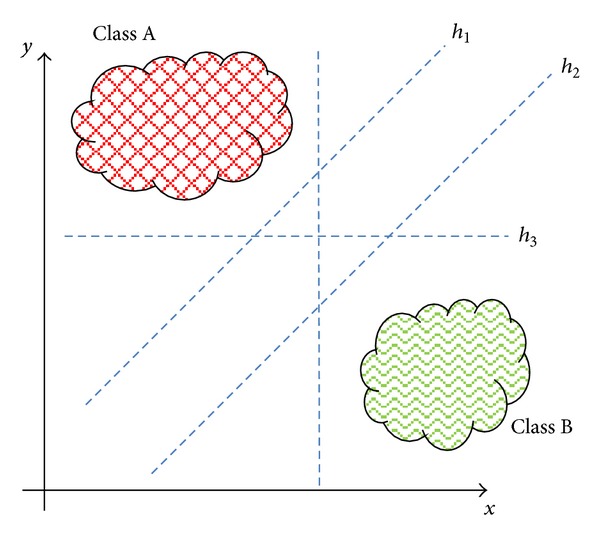
Linearly separable classification.

Intuitively, it seems obvious to come to the conclusion that when solving a linear classification problem, there is a high probability of obtaining several solutions which could correctly classify the information, as shown in Figure 2.

**Figure 3:**
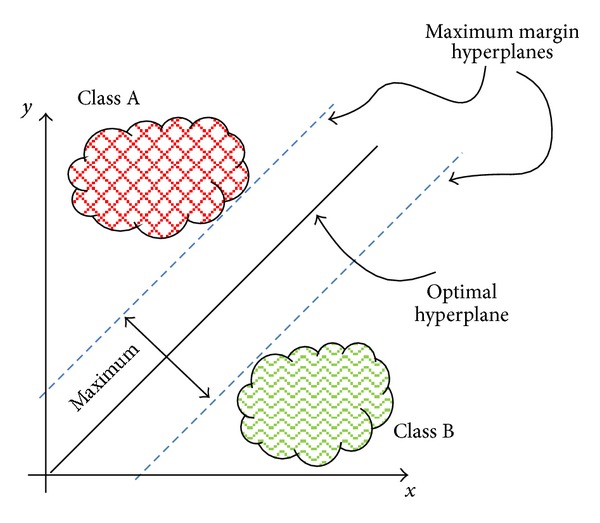
Linearly separable classification; hyperplane and separation margin.

Therefore, the question to be answered is which of the alternatives is the ideal one? In his studies, Vapnik answered this question by defining the concept of optimal hyperplane. “A hyperplane is said to be optimal if it maximizes the margin over all hyperplanes (see Figure 3) [61].

Once defined the concept of optimal hyperplane, and after carrying out several studies, it was observed that the hyperplane could be defined only if considering certain data from the training set. These characteristic points are called “support vectors,” and they are those instances of each class which are closest to the hyperplane with maximum margin.

However, in most of the existing problems, the data are not linearly separable, so that the implementation of the above-mentioned process does not achieve a good result. To solve this drawback, we should tackle these problems with different strategies, thus achieving a linear separation but in a different space. To this end, a transformation of input variables is performed in a dimensional space greater than the one to which they belong (the greater dimensional space being a Hilbert space):

(1) $$\begin{array}{c} x:{\mathbb{R}}^{n}\mapsto \varphi \left(x\right):ℋ. \end{array}$$

The next step is to find a hyperplane (actually a scalar product of vectors that can be expressed as a function of the input space *x*) in this new dimension that allows separating the data linearly. The result of this scalar product is called kernel, and the most common ones are as follows:

- linear kernel: *K*(*x*_*i*_, *x*_*j*_) = *x*_*i*_^*T*^ *x*_*j*_,
- Gaussian kernel: *K*(*x*_*i*_, *x*_*j*_) = exp⁡(||*x*_*i*_ − *x*_*j*_||^2^/2*σ* ^2^),
- polynomial kernel (order *n*): *K*(*x*_*i*_, *x*_*j*_) = (*x*_*i*_^*T*^ *x*_*j*_+1)^*n*^.

In general, a kernel is any function *K*(*u*, *v*) that verifies Mercer's theorem [62], that is, any function that verifies

(2) $$\begin{array}{c} \int_{u,v}K\left(u,v\right)g\left(u\right)g\left(v\right)du\;dv>0\\ \text{for}\;\text{every}\;g\left(\cdot \right)\text{function}\;\text{of}\;\text{integrable}\;\text{square}. \end{array}$$

Given the above, if the transformed function gives rise to a linearly separable space search, Vapnik and Chervonesky [63] showed that maximizing the separation margin between classes is equivalent to the minimization of the Euclidean norm of the weight vector. That is, considering the approximation of the set {*x*_*i*_, *y*_*i*_}con*x* ∈ ℝ^*n*^, *y* ∈ ℝ by the linear function *f* : *X*⊆ℝ^*n*^ → ℝ,

(3) $$\begin{array}{c} f\left(x\right)=w^{T}x+b=\;\overset{n}{\underset{i=1}{\sum }}w_{i}x_{i}+b, \end{array}$$

where *w* ∈ *ℜ* ^*n*^ (weight vector) *yb* ∈ *ℜ* ^*n*^ (vector of bias) are the parameters which define the hyperplane, as the lowest training and complexity error is obtained looking for the minimal *w* ∈ *ℜ* ^*n*^.

**Figure 4:**
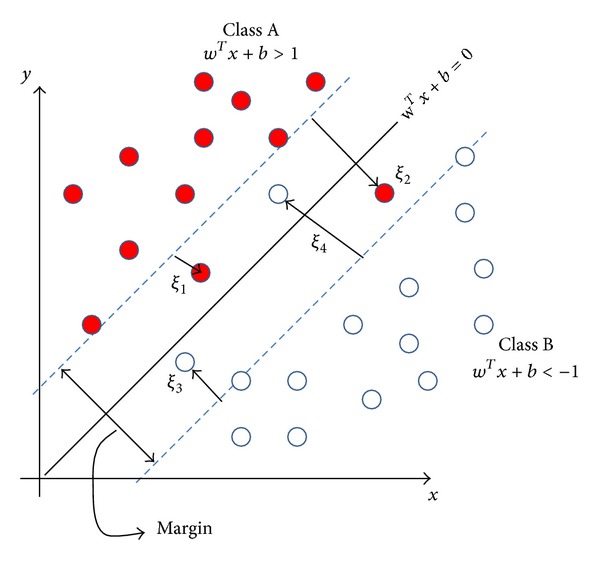
Slack variables.

But what happens when some datum is still not linearly separable in this new dimension? As shown in Figure 4, in this case the solution is to introduce a new set of slack variables: *ξ*_*i*_, *i* = 1,…, *N*, representing an estimate of the error on the optimal hyperplane.

Therefore, the problem leads to the search of a classification function *f*(*x*) that minimizes the sum of these losses reflected by the slack variables.

In this case, the function to be minimized would be as follows:

(4) $$\begin{array}{c} \Phi \left(w,\xi \right)=\frac{1}{2}w^{T}w+C\overset{N}{\underset{i=1}{\sum }}\xi_{i}, \end{array}$$

where *C* is a variable empirically specified by the user with the aim of controlling the tradeoff between the model complexity and the number of not separable data. More specifically, the greater is the value of this parameter, the higher is the assigned penalty to errors. Depending on the value of *C*, the margins of a boundary decision will vary their forms. As a result, we can conclude that the higher is the value, the narrower is the margin and the lower is the classification error in the training phase. On the contrary, the wider is the margin, the higher is the classification error in the training phase.

## 4. Proposed System

The method proposed in this work is based on creating a hybrid model that combines a GA and support vector machines, with the aim of classifying samples before selecting the minimum number of significant variables.

The GAlib library [64], developed by Matthew Wall in 1996 and last modified in 2007, was used to encode the genetic algorithms. Similarly, the WinSVM code was used to implement vector machines, a code developed by Sewell [65]. WinSVM provides as output a mean squared error (MSE) to measure the distance between the samples incorrectly classified on the optimal hyperplane. Thus, the obtained MSE value will be deterministic; hence, this will be one of the criteria which will be subsequently considered when comparing different executions.

According to those mentioned in the previous section, the SVM are extremely useful when trying to make dichotomous classification of data, that is, to distinguish between two classes. However, this idea can be generalized to identify, among a set of *n* categories, to which a certain datum belongs. In this work we have chosen to raise the following approach: considering that in the total data set *n* categories (*C*_1_, *C*_2_, …, *C*_*n*_) can be defined for each possible *C*_*i*_ category existing in the input set, and an SVM is created. This latter will try to distinguish whether a datum belongs to the given *C*_*i*_ category or to the remaining set. Finally, to determine to which specific category each datum belongs, we have simply implemented all the defined SVM and we have selected that output which indicated a greater degree of belonging to a particular class (e.g., that in which the MSE value is lower).

With the aim of creating the above-mentioned hybrid model, we have modified the traditional operation of the GA in such a way that it could generate a population of individuals of varying length [66]. To this end, we have implemented an initialization function that will be responsible for, firstly, either performing a random generation or with a predetermined size, of the length of each individual making up the population of the genetic algorithm and, secondly, initializing the value of each gene. For this purpose, we have selected from the total set of variables a subset of random size so that, if the variables generated are different, the subset will be assigned to the individual. Otherwise, a new random combination will be generated until the condition stipulated is met. This procedure is repeated until all individuals in the GA population have an assigned subset. Therefore, the result is a set of individuals as shown in Algorithm 1.

Once the GA population is created, it will be assessed by a fitness function.

**Figure 5:**
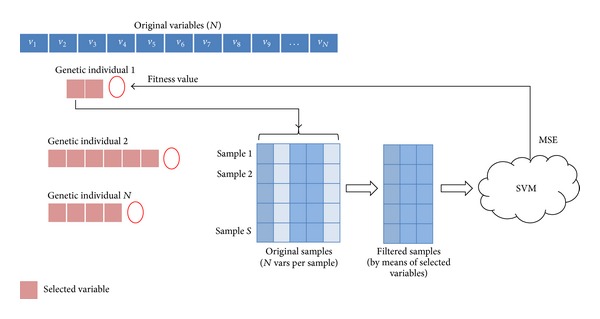
Evaluation of the genetic individuals.

In this paper, we suggest the use of the SVM as a fitness function of the genetic algorithm. Thus, for each individual, and depending on the indicated positions, a training and validation set is created from the initial data. These sets will be applied to the SVM which, once the prediction is made, will yield a mean squared error (MSE) to be used as a scale to determine the fittest individual (see Figure 5).

Nevertheless, when the information refers to a nonbinary (or dichotomic) classification, it is necessary to modify a small aspect. It will be supposed that the problem has *N* classes {*c*_1_, *c*_2_, …, *c*_*n*_}. In this case an individual SVM is applied, using the variables specified by the genetic individual, to discriminate between each of the classes c_i_ and the rest a MSE being obtained. In this case of multiclass problems, the value of kindness of the individual will be the sum of the MSE obtained for every classification.

This choice of the fitness function allows, among other advantages, a repeatability of results, which hardly ever applies to other techniques such as artificial neural networks, used in similar problems tackled in previous studies [13, 67, 68].

Therefore, this paper is a step forward regarding previous studies on selection of variables in an experimental field on which numerous tests have been carried out. Still, as shown below, the obtained results significantly improved the previous ones.

## 5. Materials

### 5.1. Data Description

Nowadays the society awareness has evolved into the need for a more and more healthy diet in order to improve the quality of life. Undoubtedly, the juice manufacturing industry, influenced by these new circumstances, has enjoyed a boom in both production and sales. However, the increasing production of these industries leads to an increase regarding the level of adulteration of their products. Consequently, the search for new methods that allow identifying the exact amount of pure juice used to produce these products has become an issue of great importance in recent years [69].

In order to prevent and detect adulteration in food, this latter must be subjected to an increasingly strict series of quality control tests. This is due to the fact that the commonly used analysis techniques have become obsolete with their development and progress. Different techniques such as HPLC (*high performance liquid chromatography*) gas chromatography, or isotope methods are too slow and relatively expensive and, therefore, they are not suitable for carrying out routine analysis. On the other hand, IR (infrared) spectroscopy provides a quick and cheap alternative, which, besides these already mentioned characteristics, provides great information about the main components of the juice.

Therefore, in order to perform testing, we have used information that allows verifying the authenticity of the apple juice quality. Using various types of apples, such as Golden Delicious, Gloster, Granny, Smith, Reineta, Royal Gala, and Starking, their juice has been extracted for subsequent centrifuging, filtering and classification using Fourier transform mid-infrared attenuated total reflectance (FTMIR-ATR). As a result, we have obtained a series of samples of diluted pure juice, which will be used to obtain the different training and validation sets.

Two sets of samples will be considered: one for samples with high concentration of juice (see Table 1) and one for samples with low concentration of juice (see Table 2). Consequently, beverages with a low concentration of juice fall within what is called energy drinks (among which soft drinks are included), while beverages with a higher concentration receive the generic name of juices. The samples made up of 20% of diluted juice (the boundary between what is considered low and high concentrations) are found in both sets.

**Figure 6:**
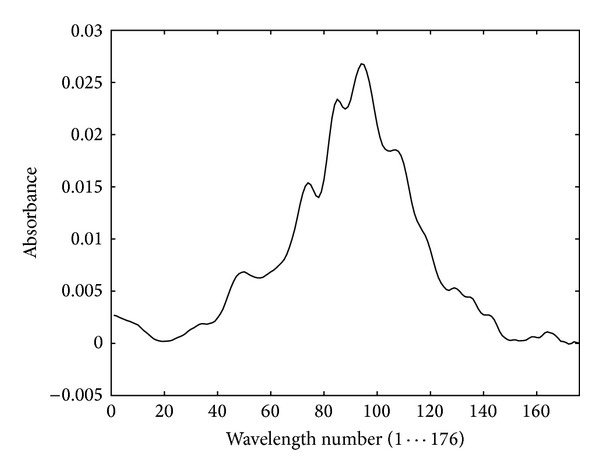
IR spectrum, specific to a sample.

All samples were characterized by means of an infrared spectroscopy. As a result of this characterization, a spectrum (as shown in Figure 6) is obtained for each sample, which represents the amount of energy absorbed (or absorbance) for a total of 176 wavelengths or variables.

The objective is to determine the amount of juice in a sample using only the information provided by this spectrum. However, using all the information of the spectrum does not lead to fully satisfactory results, as shown below. That is why the need for applying a process of selection of variables is arisen. This would have two obvious advantages. On the one hand, it is time-saving (both when obtaining the spectrum and in the subsequent classification, since a smaller amount of information is involved). On the other hand, and perhaps most importantly, the expert is provided further information about which part of the spectrum, that is, what specific type of sugar (fructose, sucrose, etc.), provides more information to carry out this classification.

### 5.2. Parameter Setting

The first experiment that should be performed before running the developed model is the choice of setting parameters for both vector machines and GA. To this end, we have applied the following procedure.

To determine the setting parameters of the SVM algorithm, the steps proposed by the author have been followed, using the “optimize” option provided by the algorithm itself. This function is based on a random generation of different combinations of the SVM parameters, which are then applied to the initial data obtaining a mean squared error (MSE) as a result of the application.

A total of 100 different combinations were generated for this purpose, and from among them, and taking as scale the training MSE, we have chosen those with the best results in the training cases. However, when performing this first test, there were no entirely satisfactory solutions (see Table 3). On the other hand, this fact is not really significant since the final aim in this phase is to determine the configuration parameters of the algorithm, not to carry out a real data classification.

Tables 3 and 4 show the three best combinations obtained from the 100 tested (for both high and low concentration), selecting as optimal the one whose training mean squared error is lower. It is noted that the penalty variable *C*, in both cases, has a relatively high value (*C* = 10,000 for high concentration and *C* = 1,000 for low concentration), leading to, as mentioned above, a considerable reduction of the error training (3.83E-07 and 5.26E-07, resp.) finally obtained.

The configuration of the genetic algorithm parameters, carried out similarly as in the case of the SVM, should be selected after performing a series of tests by varying their value. From the range of tests, the configuration shown in Table 5 was taken as optimal.

## 6. Results and Discussion

First, to allow the comparison of results, a reference model should be established. In this case, we have chosen the original set of 176 variables provided by the IR spectrometer to build different classification models starting from the former. More specifically, we have chosen some of the most widely used models in the field of analytical chemistry with this type of data (partial least squares: PLS, SIMCA or potential functions), together with a model generated from the use of ANN and another one using SVM for the classification. Tables 6 and 7 show the results obtained with each of these models.

At first, given the great performance offered by the ANNs, we had chosen this technique to guide the search for the GA. However, as discussed below, they have the great disadvantage of the repeatability of results, which leads to the fact that the variables selected as the most relevant are different in each iteration.

On the other hand, the results obtained in this first test using SVM can seem discouraging. However, this can be explained according to the above classification criteria. Thus, for a given concentration, SVM are applied for each type of concentration (20%, 25%,…) obtaining a good or bad classification result regarding the individual classification. A good result in the final classification of the sample will be taken into account only if the datum is always assigned (i.e., for all the developed SVM) to the right category. For example, a classification of a datum with 20% juice is considered correct only if the SVM that discriminate the category of 20% allocate it to that category, while the remaining defined SVM (each for the discrimination of one category) always assign the datum to the class of “other categories” (among which there is one of 20%). If, on the contrary, the sample is incorrectly characterized by a SVM, then the end result will be regarded as wrong.

As mentioned earlier, the results achieved in this test using the SVM are not satisfactory. Undoubtedly, any of the techniques employed for performing the classification (ANN, PLS, …) achieve better results than the SVM.

Previous studies, based on the use of a tool widely employed in the study of chemical data as Procrustes rotation [70], have allowed establishing that the information provided by two of the original variables would be enough to categorize the data. The existence of overinformation and the difficulty that this involves when generalizing may be some of the causes of the poor results provided by SVM. Thus, selecting the most important variables from their original set we may obtain similar or better results than using all the data. This is due to the fact that, in many cases, not all the original variables have the same influence, or they contain redundant information, when determining the actual content of juice in a sample.

Therefore, a second criterion of comparison or a reference model is established, in this case one consisting of the classifications made only from the two significant variables previously selected by Procrustes (specifically, the variable 94 and the variable 95).

The results for each of these models are shown in Tables 8 and 9.

In this case, the proper operation of SVM is shown with a smaller version of the data set provided by IR spectroscopy, reducing the number of errors obtained so far in other studies. Therefore, its use as a measure of goodness seems feasible when guiding the implementation process of variable selection by means of GAs.

As previously mentioned, the initial goal consists of determining by GAs a series of sets obtained from the original variables that allow the correct classification of a sample. Furthermore, by not providing a single solution—as does Procrustes Rotation—further information is provided to the expert about the importance of different regions of the spectrum. First, the GA is configured to generate individuals made up of only 2 genes, each of them representing each of the variables to be considered for performing the classification.

In previous studies [13, 17, 67, 71], due to the versatility of ANNs, this technique has been chosen as the evaluation function used by the GA. Thus, the GA selects variables considered as *significant*, which will be provided as inputs to an ANN that is responsible for classifying the samples according to their amount of juice. The MSE obtained in the training process of the ANN is used as fitness to determine the goodness of each individual gene.

Tables 10 and 11 show the best results obtained with this technique after various tests and configurations. We can observe that the results improve significantly compared to those obtained when classification is made according to the variables selected by Procrustes Rotation.

However, this approach raises a problem. Due to random initialization process of an ANN, the obtained results are hardly repeatable. To solve this problem, this paper proposes the use of SVM as a fitness function employed to guide the search for the GA. On this basis, we propose two approaches for defining the fitness function. On the one hand, the training mean squared error (MSETrain) is used as a fitness function, while on the other hand, the sum between the training MSE and the validation MSE (MSETrain + MSETest) is used as a fitness function.

Depending on the selected option as a fitness function, we have obtained the results shown in Tables 12 and 13, which include the four best solutions reached during the execution of the hybrid system using the two possibilities raised here as a fitness function. They show the predictive variables, as well as the errors made during the training and validation phases. As it can be seen by analyzing the results, regardless of the fitness function chosen, the obtained results are not only improved when the classification is done starting from the variables selected by Procrustes, but they are comparable to those obtained from the initial data set (unlike other techniques). In addition, compared to the use of ANN, we can count on the advantage offered by the repeatability of results.

Comparing these results with those previously shown (Tables 10 and 11), we can observe that the results in the training phase are very similar, regardless of the use of ANN or SVM in the evaluation function. However, the results obtained by SVM on the validation and testing data significantly improve the ones obtained with ANN, coming to the conclusion that greater generalization ability is achieved.

**Figure 7:**
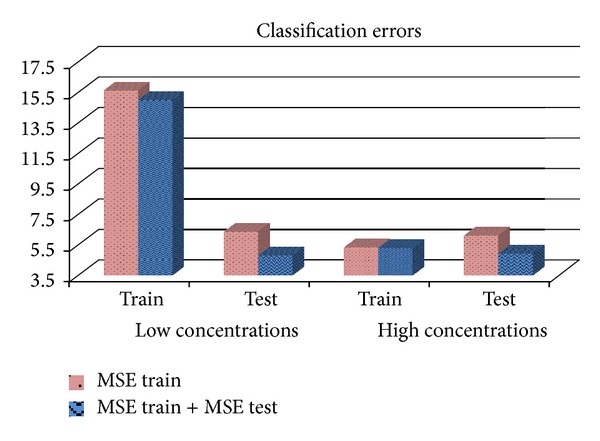
Classification errors according to the used fitness function.

On the other hand, according to the results presented in Tables 12 and 13, one can sense that the use of the sum of both MSE (MSETrain + MSETest) as a fitness function of the hybrid system is able to reduce the number of errors made during the classification. The graph shown in Figure 7 performs a comparison between the two implemented functions. The data displayed correspond to the average of errors made during the SVM classification of all the executions carried out with the hybrid system. A slight superiority is observed when using the sum of both MSE as a fitness function, especially in the validation phase.

## 7. Conclusions

This paper has proposed the development of a hybrid model combining GAs and SVM. The aim pursued by using this approach is the identification, from the initial data set, of the smallest number of variables possible that allow determining optimally the amount of juice within a sample. To this end, the method proposed herein uses a GA that employs SVM as a fitness function. This would lead to the generation of a fast, effective model that is capable of reproducing the data obtained.

With the aim of demonstrating the effectiveness of the proposed system, a series of experiments have been raised, with their corresponding validation tests. Observing the values obtained, we can come to the conclusion that the system improves significantly the results obtained so far with other classification methods.

As noted above, SVM are useful and effective when performing prediction data with a margin of error, previously cited as *ξ*. This evaluation mechanism has the undeniable advantage of the repeatability of results. Thus, each set of variables will always have the same prediction system associated with the same fitness level, contrary to what would happen in the case of using ANN as a measure of goodness for genetic individuals.

## Figures and Tables

**Algorithm 1:**
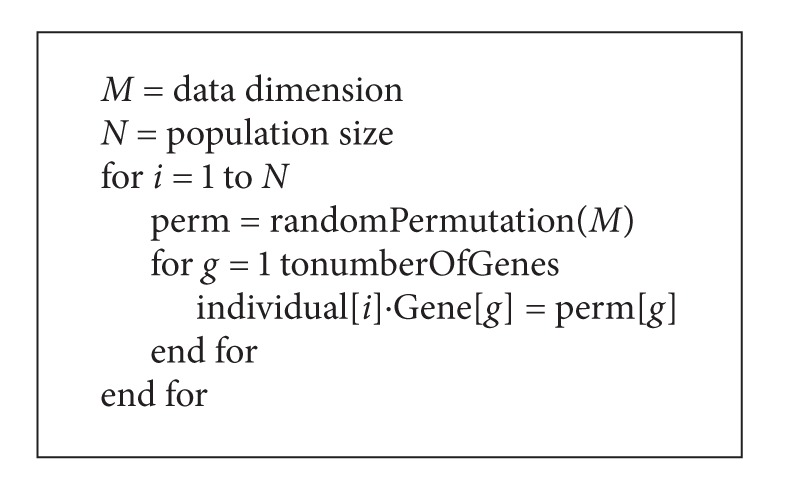


**Table 1:** Samples with high concentration.

Range: 20%–100%			
%	Training	Test	Commercial
20%	20	6	
25%	19	18	2
50%	16	13	
70%	14	1	
100%	17	6	19

Total	**86**	**44**	**21**

**Table 2:** Samples with low concentration.

Range: 2%–20%			
%	Training	Test	Commercial
2%	19	1	
4%	17	1	
6%	16	13	
8%	22	6	
10%	21	6	1
16%	20	6	1
20%	19	6	

Total	**134**	**39**	**2**

**Table 3:** Setting of SVM parameters: samples with high concentration.

	SVM parameters			MSE train
	*C*	Epsilon	Radial	
1°	10000	0,000001	5	3,83*E* − 07
2°	10000	0,0001	2	4,49*E* − 07
3°	10000	0,0001	2	4,49*E* − 07

**Table 4:** Setting of SVM parameters: samples with low concentration.

	SVM parameters			MSE train
	*C*	Epsilon	Radial	
1°	1000	0,000001	2	5,26*E* − 07
2°	1000	0,000001	10	5,95*E* − 07
3°	1000	0,0001	2	5,95*E* − 07

**Table 5:** Genetic algorithm configuration.

(i) GA: simple	(i) Selection: wheel-roulette
(ii) Number of individuals: 100	(ii) Number of generations: 100
(iii) Crossover rate: 90%	(iii) Crossover: uniform
(iv) Mutation rate: 10%	(iv) Mutation: uniform

**Table 6:** High concentration: classification errors using 176 variables.

	Training (86 samples)	Validation (44 samples)	Commercial (21 samples)
PLS	11	5	1
SIMCA	15	12	1
Potential functions	4	6	0
ANN	0	1	0
SVM	0	39	21

**Table 7:** Low concentration: classification errors using 176 variables.

	Training (134 samples)	Validation (39 samples)	Commercial (2 samples)
PLS	29	11	0
SIMCA	19	14	0
Potential functions	4	9	0
ANN	0	4	0
SVM	0	33	1

**Table 8:** Classification errors using variables selected by Procrustes rotation. High concentration.

	Training (86 samples)	Validation (44 samples)	Commercial (21 samples)
PLS	6	6	1
SIMCA	44	27	8
Potential functions	5	6	0
ANN	6	8	0
SVM	5	3	3

**Table 9:** Classification errors using variables selected by Procrustes rotation. Low concentration.

	Training (134 samples)	Validation (39 samples)	Commercial (2 samples)
PLS	29	9	0
SIMCA	36	17	0
Potential functions	29	13	0
ANN	28	17	2
SVM	13	4	1

**Table 10:** Classification Errors using GA + ANN: high concentration.

High concentrations			
Selected variables	Train (86 samples)	Test (44 samples)	Commercial (21 samples)
[52, 141]	4	8	1
[102, 129]	10	14	2
[23, 67]	5	16	1
[18, 120]	3	12	2

**Table 11:** Classification errors using GA + ANN: low concentration.

Low concentrations			
Selected variables	Train (134 samples)	Test (39 samples)	Commercial (2 samples)
[52, 141]	19	17	2
[102, 129]	10	14	2
[23, 67]	5	16	1
[18, 120]	3	12	2

**Table 12:** Classification errors using GA + SVM: high concentration.

Selected variables	Train (86 samples)	Test (44 samples)	Commercial (21 samples)
MSE train			
[4, 69]	5	5	2
[81, 84]	9	5	2
[68, 28]	4	6	2
[91, 101]	4	6	1

MSE train + MSE test			
[9, 105]	5	3	3
[9, 113]	5	4	9
[34, 101]	4	5	11
[86, 104]	5	5	4

**Table 13:** Classification errors using GA + SVM: low concentration.

Selected variables	Train (134 samples)	Test (39 samples)	Commercial (2 samples)
MSE train			
[1, 107]	15	5	1
[5, 91]	16	5	1
[2, 107]	17	5	1
[11, 97]	14	6	1

MSE train + MSE test			
[5, 73]	13	4	1
[27, 90]	14	4	1
[2, 94]	15	4	1
[27, 88]	14	5	2
